# The pan-Canadian Tiered Pricing Framework and Chinese National Volume-Based Procurement: A comparative study using Donabedian’s structure-process-outcome framework

**DOI:** 10.7189/jogh.13.04137

**Published:** 2023-11-10

**Authors:** Quan Wang, Siqi Liu, Zhijie Nie, Zheng Zhu, Yaqun Fu, Jiawei Zhang, Xia Wei, Li Yang, Xiaolin Wei

**Affiliations:** 1 School of Public Health, Peking University, Beijing, China; 2 Brown School, Washington University in St. Louis, St. Louis, Missouri, USA; 3 Center of Health System and Policy, Institute of Medical Information & Library, Chinese Academy of Medical Sciences & Peking Union Medical College, Beijing, China; 4 Institute of Health Policy, Management, and Evaluation (IHPME), University of Toronto, Toronto, Ontario, Canada; 5 Department of Health Services Research and Policy, London School of Hygiene & Tropical Medicine, London, England, UK; 6 Dalla Lana School of Public Health, University of Toronto, Toronto, Ontario, Canada

## Abstract

**Background:**

Generic drugs have been seen as a potentially powerful way to alleviate the financial burden on patients and health care systems. Two strategies for achieving rational prices of generic drugs are tiered pricing framework and pooled purchasing power. We compare the pan-Canadian Tiered Pricing Framework (TPF) and the Chinese National Volume-Based Procurement (NVBP) as comparators to explore the similarities and differences between the two mechanisms and summarise lessons for other jurisdictions.

**Methods:**

This comparative study applies Donabedian’s structure-process-outcome framework to systematically analyse the macro contexts, procedures, and long- and short-term results of each pricing mechanism, and the interactions between them.

**Results:**

Structure: TPF is an upstream initiative aimed at lowering the prices of generic drugs and increasing coverage and price consistency. NVBP is a downstream national initiative prioritised for reducing drug prices to achieve value-based purchasing. Process: By associating the number of manufacturers with price cuts, TPF leaves the choice to manufacturers to decide if they want to enter a specific market. In contrast, the Chinese government determines NVBP list and has the authority to choose manufacturer(s) with the lowest price(s). TPF provides clear price information to potential suppliers with unclear order quantity. The NVBP drug price is determined by tendering, while procurement volume is clear and massive. Outcome: The effectiveness of TPF and NVBP is similar, with both achieving a 53% price cut. Both TPF and NVBP experienced efficiency improvement since their establishment, with 98 and 86 drugs priced per year. By comparing 60 drugs covered by both programmes, the NVBP price is 57% of that of the TPF counterpart on average (1.1 to 301.6%), by purchase power parity.

**Conclusions:**

The tiered pricing scheme is feasible in regions with a stable and mature pharmaceutical market, typically seen in high-income countries, while tendering is more workable in low- and middle-income countries where the pharmaceutical market is weak and unstable. Experience in the two countries shows that a coordinated pricing mechanism involves many piecemeal interactive problems, which a sophisticated system with a robust long-range plan may address better.

In recent years, rising health care costs, especially those related to pharmaceuticals, have become a major concern for many countries [1,2]. In low- or middle-income countries (LMICs), medicine expenditures can account for up to 60% of total health care spending and most of the population pays for it out-of-pocket [1]. Similarly, medicine expenses account for an average of 18% of total health care expenditures in high-income countries [3]. Addressing drug pricing is crucial to mitigate the financial burden on health care systems and improve population health.

Generic drugs have been proposed as a potential solution to reduce health care costs, as they are often more affordable than their originator drugs [4,5]. However, mark-ups on generic drugs are still common, hindering universal health coverage [4,6]. Various pricing mechanisms, such as linking the number of generic drug manufacturers with price cuts and pooling bulk purchasing power to negotiate prices with manufacturers, have been implemented in many countries [7-9]. The former pricing mechanism begins with the providers; the greater the number of generic drug manufacturers, the lower the price. Pharmaceutical manufacturers may choose to enter the market when they expect to earn profits at the prevailing price. Entry stops once the marginal manufacturers earn no profit at the price that would prevail after they enter [10]. The latter strategy that might effectively work to motivate firms to provide price concessions is pooling bulk purchasing power from multiple payers into an alliance to negotiate drug prices with manufacturers [11]. The nature of this approach is tendering which will award huge enough contract(s) to the lowest-price manufacturer(s). Many LMICs in Western Pacific Region or Latin America implement this strategy, like India, China, Malaysia and Vietnam [12,13].

Successful drug price negotiations have reported several benefits, including improving the affordability of medicines, enhancing equity of access, contributing to administrative efficiency, eliminating duplication of efforts, and shortening the timeline for drugs to enter formularies [12]. However, there is a paucity of rigorous research that compares the two approaches to generic drug pricing mechanisms under various contexts in significant detail, which makes it difficult for policymakers to obtain recommendations and decide on their next steps. Therefore, there is a need for more rigorous research to compare and evaluate the effectiveness of different pricing mechanisms, to inform policy decisions and improve access to affordable medicines.

The Tiered Pricing Framework (TPF) by the pan-Canadian Pharmaceutical Alliance (pCPA) and the National Volume-Based Procurement (NVBP) in China are two attempts of the above two approaches to achieve rational generic drug prices. This study aims to provide a comparative analysis of the pan-Canadian Tiered Pricing Framework and the NVBP, to better understand the similarities and differences between these approaches, their impacts on stakeholders, and the lessons that can be learned from these initiatives for other jurisdictions. The findings of this study may contribute to the development of more effective and efficient drug pricing policies and have important implications for health care systems and populations worldwide.

## METHODS

### Conceptual framework

Donabedian’s framework provides a solid basis that could systematically guide the research and evaluation of the two policies and comprehensively probe all the elements involved. According to the framework, the analysis path could be categorised into three variables: structure, process and outcome (SPO).

Structure is defined as the setting where a policy occurs. For this study, we specifically define the structure component as the macro context of the health care system, including its design, reform objectives, previous pricing cut attempts and policy objectives. We also consider the type of negotiated drugs and the key players involved in policy development, such as promoters and stakeholders, as they are important factors in understanding the specific policies and why they were created. Additionally, the historical and socioeconomic context of either country will be considered as an important aspect of the structure component, as it can shape the environment in which policies are introduced and implemented.

Process refers to each clear step performed by the structure as the overall policy implementation progress. Process here involves all concrete procedures of the TPF and NVBP from beginning to end, meanwhile, the detailed rules in each procedure will be articulated as well, such as initial leadership of the policies, drug inclusion criteria, obligations and positions of each institution, final procurement and drug insurance listing.

Outcome ultimately describes the explicit results demonstrated by the antecedents of structure and process. In the outcome of this analysis, the comparative results of negotiation activities, price, and price reductions will be presented. The potential impact of these policies on key stakeholders, such as patients, health care providers, and pharmaceutical companies, will also be examined.

There is a directionality effect among the three dimensions in the linear model, and each component will be directly impacted by the antecedent one either positively or negatively. In the next sections, these three components are equally explored to provide a comprehensive and nuanced analysis of the TPF and NVBP policies.

### Data collection and synthesis

We conducted a rigorous literature search to gather relevant information on the topic. Our comprehensive search strategy included the major databases of MEDLINE, Web of Science and Embase, as well as screening of relevant grey literature using the Grey Matter Checklist. We used a combination of Medical Subject Headings terms and text-words in the following concept areas: Tiered Pricing Framework, pan-Canadian Pharmaceutical Alliance, Volume-based Procurement, and 4 + 7. Additionally, we manually searched the reference lists of full-text articles to identify any additional relevant studies. A detailed description of our search strategy and data extraction process is presented in Appendix S1 in the **Online Supplementary Document**.

The materials used for analysis included not only published studies but also authors' opinions and ideas that were generated through academic forums, in-depth contemplation, interaction with policymakers and years of experience in drug policy and health economics. We utilised inferential, deductive and counterfactual reasoning to test proposed hypotheses and examine underlying reasons for our findings. This approach allowed us to go beyond the available evidence and draw upon a wide range of knowledge and expertise in the field.

### Comparative analysis

#### Structure

##### Tiered Pricing Framework Structure

The organisational structure of the Canadian health care system is highly decentralised. Rather than having one national health plan, the 13 provinces/territories' governments bear the individual responsibilities for the management, organisation and delivery of health care services for their residents. Under this system, all Canadians have the opportunity to access medically essential services without any out-of-pocket fees [14]. The goals of health care system reform in Canada include a pan-Canadian system of drug coverage and structural reforms to improve efficiency [15]. However, the national legislation has no provision for mandatory universal drug coverage. Each province/territory has evolved its unique systems of public drug insurance within their jurisdiction, which has led to Canada becoming the only developed country with universal health coverage that excludes drug coverage [16]. The population covered and the available drugs vary significantly across Canada.

Given the high drug costs but a paucity of universal drug insurance, the province/territory drug plans have increasingly strong incentives to negotiate price discounts with pharmaceutical companies. Therefore, the pan-Canadian Pricing Alliance was created in August 2010 to negotiate price discounts with pharmaceutical companies for public drug plans across all provinces/territories and federally administered.

The pCPA member jurisdictions include public drug plans and/or cancer agency participation. Although the federal and private drug insurance plans are not participating, the latter has expressed a willingness to participate in pCPA [17]. Despite the hodgepodge of public drug plans in Canada that vary in each province/territory, the federal government still has the stewardship of pharmaceutical approval and regulation. As for the governance of the pCPA, province/territory Deputy Ministers of Health oversee pCPA initiatives and appoint a chair of the Governing Council, which leads the pCPA and the pCPA office. They meet twice annually and on an “as-needed” basis to engage in knowledge sharing on jurisdictional priorities, issues and concerns that impact the Canadian health system, and identify and direct initiatives that require collective leadership and action [18]. Representatives selected from Governing Council and Drug Plan Leads assemble the Management Committee, which executes the strategic direction of the Governing Council, addresses issues or disputes that may arise in negotiations, provides direction and assistance to Drug Plan Leads, and provides managerial direction to the pCPA Office. Drug Plan Leads are Operational leaders for the implementation of pCPA initiatives for each jurisdiction and they provide jurisdictional perspectives in the negotiation process and work with the pCPA office to improve the efficiency and effectiveness across jurisdictions.

**Figure 1:**
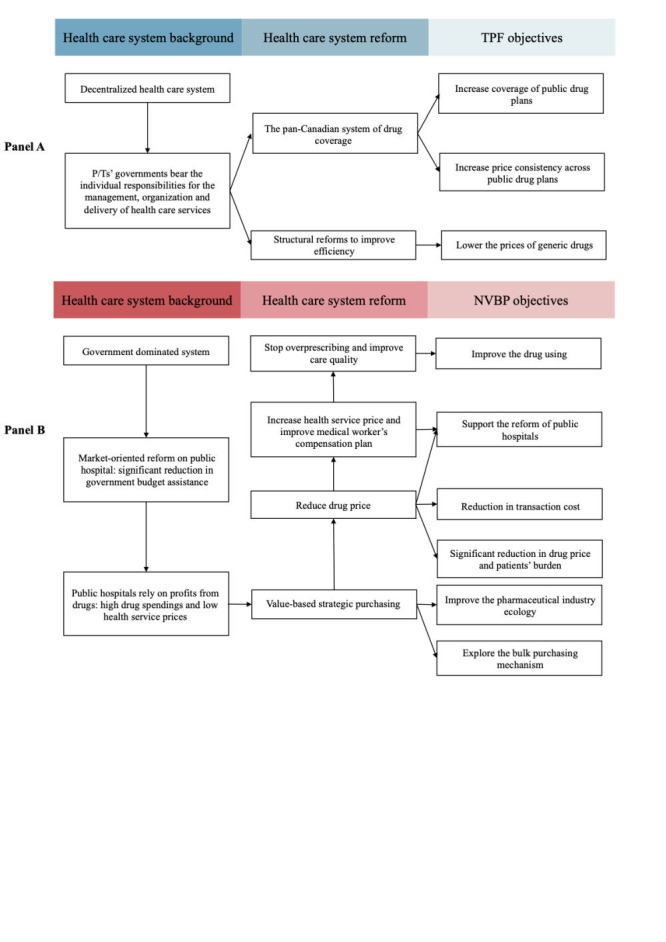
The main structure information of Tiered Pricing Framework (TPF) and National Volume-Based Procurement (NVBP). **Panel A.** Structure information of TPF. **Panel B.** Structure information of NVBP.

All drug price negotiations are classified into two categories: the pCPA negotiation for brand name drugs and TFP for generic drugs, the former of which are prioritised products by pCPA. As stated by itself, the main objectives of TFP are to lower the prices of generic drugs and to increase coverage and price consistency across public drug plans [19]. The relationship between Canada’s health care system, reforms and TPF has been shown in **Figure 1**, panel A.

##### National Volume-Based Procurement Structure

The Chinese health care system has undergone several rounds of administrative reforms aimed at decentralisation, but the role of the central government remains dominant in health care decision-making and legislation.

The Open and Reform policy in the late 1970s led to fiscal neglect of public hospitals [20] and they became increasingly dependent on profits from overprescribing and selling expensive medicines in balancing revenue and expenses. A high proportion of pharmaceuticals occupies spending that should be allocated to health services. For instance, the fee for medical consultation in China is usually lower than 10 Renminbi (RMB) (about two Canadian dollars). The prices of health services provided by doctors and nurses are much lower than their values, which motivates them to overprescribe for profits. Therefore, one of the core objectives of the Chinese health care system is to adopt value-based strategic purchasing to align incentives with the needs, quality, and outcomes of patients. By reducing costs spent on drugs, more money can be paid to doctors and nurses for health services. Hence, the government can reform the pricing mechanism of health services and the medical worker’s compensation plan, which should redirect the motivation from prescribing pharmaceuticals to providing high-quality care. The cornerstone of the whole reform relies on how to successfully reduce drug prices.

In 2018, China began to implement the National Volume-Based Procurement programme to encourage large-scale group purchases of high-quality drugs and improve the security of the drug supply in the country. The first round of NVBP involved four provincial cities and seven sub-provincial cities, known as the “4 + 7 scheme”. In the following year, all provincial entities in mainland China joined the NVBP programme.

A working group was created to organise the NVBP and led by the State Council, which is the highest executive body in China. Representatives from the national health payer, regulator, and administrator participate in the process. The National Healthcare Security Administration (NHSA), a newly formed agency in 2018, administers most of the NVBP programme. It has the authority for pricing and procurement of drugs and the stewardship of health insurance payments across the country. The National Medical Products Administration (NMPA) is responsible for regulating quality evaluation and drug assessment in the decision-making process and conducts the Generics Consistency Evaluation (GCE), a mandatory bioequivalence test introduced in 2015 to review the quality and efficacy of China's domestically produced generic drugs in comparison with their brand-name counterparts. The National Health Commission (NHC) facilitates the programme by introducing policies that motivate the purchasing and prescription of selected drugs and manage the behaviour of health providers [21]. Under the working group, the Joint Procurement Office (JPO) supervises the bidding-tendering and procurement processes, and the Sunshine Medical Procurement All-In-One (a local drug, diagnostic, and device procurement agency in Shanghai) supports the daily work of the JPO. The JPO is composed of representatives who serve as deputy directors from the local governments of the jurisdictions involved. The directors of the JPO are elected by these deputy directors. These representatives act as agents of public hospitals in their respective jurisdictions, responsible for organising, conducting and overseeing the NVBP process.

According to government files, the main objectives of the NVBP programme include a significant reduction in drug prices and patients' burden, a reduction in transaction costs and improvement of the pharmaceutical industry ecology, improvement of drug use, support for the reform of public hospitals and exploration of the bulk purchasing mechanism. The relationship between the Chinese health care system, reforms and NVBP is shown in **Figure 1**, panel B.

#### Structure comparison

The dimension of structure is complex and shows obvious variations in terms of practice context and type. First, when looking at the background, we found that the Chinese health care system is still in active transition with several clear reform objectives, whereas the health care system in Canada is relatively stable. While managing to lower the price of generic drugs is a shared objective of both TPF and NVBP, different health care system structures and histories have endowed these two programmes with different missions. According to the Health Canada Act of 1984, the province/territory governments are responsible for health insurance in their jurisdictions. As a consequence, although all residents of Canada are eligible for publicly funded drugs, the coverage varies considerably across the country [22]. Therefore, increasing coverage and price consistency across public drug plans are also important objectives of TPF. As for the NVBP, other main objectives include exploring rational generic drug pricing mechanisms and improving the pharmaceutical industry ecology. One possible reason is pharmaceutical market maturity: Canada has a mature pricing mechanism based on the principles of the market, with massive written and unwritten rules, while the Chinese governments (both central and local) are still exploring a rational pricing system for drugs. In fact, during a very long time in the past, even current, drug pricing in China is not based on market activity but on obscure internal negotiations between medical institutes and pharmaceutical companies, where massive bribes grow. Besides, the reimbursement health insurance system in China leads to another significant difference: NVBP also focuses on the patient’s financial burden. It is worthy of note that this is not equal to lower drug prices which is merely a part. Impeding overprescription and using generics as an alternative to brand-name drugs can effectively reduce the cost pressure on both patients and the health insurance system.

As we noticed, aligned with their specific political conditions, the most significant divergence between the pCPA and the NVBP is the leadership. The pCPA is initiated at the province/territory level, while the NVBP is nationally organised. Under the pCPA, what is proved to be a hurdle is the patchwork of public drug plans in Canada. The provinces/territories are accountable for individual revenue streams, demographics, political priorities, government budgets and “pressures”; they might therefore undoubtedly come to the negotiation table with varied concerns and purposes [23]. Subsequently, although members commonly share the pricing results, likely, the costs saved are not equal among all provinces/territories. For instance, incidence rates of chronic obstructive pulmonary disease in the province of Nova Scotia are much higher than the national average (7 vs. 4.2%) [17]. Drug price deduction is more cost-effective and offers better societal value in a province with a high number of cases than if listed in other provinces. Federal decentralisation in Canada might increase the difficulty of cross-regional corporations to some extent, like the difficulty to gather purchasing power we mention above. The impact of federal decentralisation is further, and one is the lack of procurement quantity expectation. The TPF only involves pricing and the following procurement is conducted by province/territory governments. Neither buyer nor the manufacturer knows the precise sales volume, which forces both of them to make the risky decision that erodes the advantage of bulk in bulk purchasing. Therefore, completing such a multi-party action would require more vigorous efforts of intergovernmental coordination and consensus. By contrast, one of the main principles of NVBP is “national organising”. Unlike previous procurement initiatives in China, NVBP is on target to restructure generic procurement in the country, so it was guaranteed a high degree of political commitment from a powerful national government, along with a bidding-tendering process at the national level, a working group directly led by the State Council and a series of unified policies in pilot areas.

Both the NVBP and pCPA programmes involve representatives from local governments, allowing for the inclusion of local interests and facilitating the acceptance of decisions by participating jurisdictions. This organisational structure ensures that local characteristics are taken into account during the design and implementation of the programmes. The fragmentation of China's health insurance system, similar to Canada's system, with coordinated medical insurance fund pools at the provincial or municipal levels, contributes to the need for incorporating local representatives into the management of NVBP. This consideration is essential due to the significant disparities in health systems across China.

#### Process

##### Tiered Pricing Framework Process

**Figure 2:**
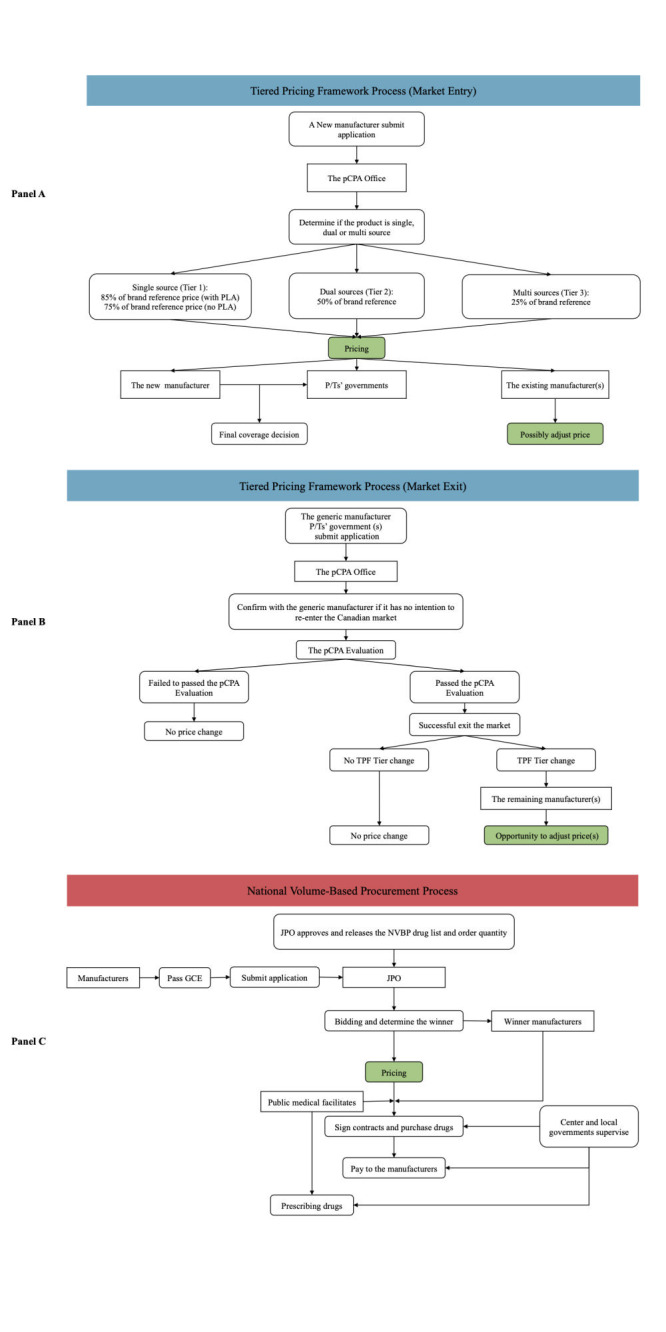
The main process information of Tiered Pricing Framework (TPF) and National Volume-Based Procurement (NVBP). **Panel A.** Market Entry Process of TPF. **Panel B.** Market Exit Process of TPF. **Panel C.** Pricing Process of NVBP.

The Tiered Pricing Framework (TPF) process for generic drugs involves market entry and market exit activities, as shown in **Figure 2**, panels A and B [24,25]. To list a generic drug on a public drug plan formulary, the manufacturer must submit an application to the pan-Canadian Pharmaceutical Alliance Office (pCPAO), which then determines the appropriate price tier based on whether the drug is a single, dual, or multi-source product. The price tiers range from 25 to 85% of the brand reference price. Once a TPF Pricing Confirmation Form is received, all competitors must adjust their prices to match the pCPA’s Calculated Unit Price. Market exit notifications trigger a similar process to assess whether the manufacturer meets the criteria for exiting the market. It is important to note that only changes in the TPF tier will trigger a price change for generic drugs, and there is currently no central process for price increase applications. A detailed process is presented in Appendix S2 in the **Online Supplementary Document**.

##### National Volume-Based Procurement Process

The NVBP process is a type of tendering conducted by the Chinese central government twice a year, covering specific drugs that meet certain requirements, such as having no less than two manufacturers in the market, being covered by the National Health Insurance List (NHIL), being clinically necessary, having a large market size and high spending. Only generic drugs that have passed the Generics Consistency Evaluation (GCE) can participate in the tendering process, which tests the quality and efficacy of China’s domestically produced generic drugs. During the bidding process, the price is the most important criterion, and the manufacturer(s) with the lowest price(s) are usually selected as the winner(s), as shown in **Figure 2**, panel C. Public medical institutions estimate the total purchase volume of the selected drugs based on past consumption, and after the final price is established, they sign contracts and purchase drugs with the final supplier independently. The procurement process is regulated by both central and local governments and 30% of the total costs are directly transferred from local health insurance bureaus to medical facilities to alleviate their financial burden. A detailed process is presented in Appendix S3 in the **Online Supplementary Document**.

#### Process comparison

Although process is typically thought to have the biggest impact on outcomes, it can also provide feedback about the structure already in place. In nature, TPF is a kind of tiered pricing scheme while NVBP is a tendering process [26], and both are significantly influenced by structural factors. As mentioned earlier, a pan-Canadian system of drug coverage is a major objective of the Canadian health care system, which consequently requires drug price consistency among different provinces/territories. Therefore, price caps and tiered pricing scheme are almost the only two workable methods to ensure price consistency. As far as we know, both were once implemented in some provinces/territories before the establishment of pCPA. From 1993 to 2013, the maximum allowable list price (MALP) was widely used as a price cap in many provincial public programmes, under which generic drug prices were capped at a fixed percentage of the respective branded product’s price [27]; in 1993, Ontario government regulated that the first generic entrant must be priced at no more than 75% of the price of the branded product, any subsequent generics at no more than 90% of the first generic price [28,29], which declined to 25% in following decades [27]. However, tiered pricing frameworks are a better choice than price caps, given the key defects of price cap programmes such as no price reduction over time [30], no available information to set price cap [31], and difficulty rationalising a “one size fits all” type of price-cap regulation [32]. Moreover, the influence of pharmaceutical market maturity is a more foundational factor that explains the institutional choice of Canada and China. The tiered pricing framework works only in countries with a relatively stable pharmaceutical market, as it is in Canada, Austria, Portugal, and South Korea [33,34]. If the number of manufacturers changes rapidly, the drug price will be unstable, which can misguide the government, patients, and the market itself. Therefore, for LMICs like China with unstable pharmaceutical manufacturer size, volume-based tendering and procurement seems a better choice. As it should be, NVBP in China is also a result of the “inertia” of previous practices, with several hospitals conducting the very first pooled procurement as early as 1993, followed by the gradual prevalence of pooled procurement by tendering at the municipal and provincial levels. In conclusion, major health care system reform objectives, pharmaceutical market maturity, and history are three key factors in policy choice in China and Canada.

The GCE policy design is unique, and influenced by certain Chinese-tailored factors. We did not find a similar practice in Canada or any other countries, even in those implemented tendering pricing mechanisms. GCE aims to test the quality and efficacy of domestically produced generic drugs in comparison to their corresponding brand-name versions. In other words, drugs that pass the GCE have identical quality and efficacy [35]. Only drugs that pass the GCE can participate in NVBP bidding. Thus, although GCE is not a legally required step in NVBP, it functions as a “step zero”. We believe there are two possible necessities for GCE. The first one still comes from the immature pharmaceutical market in China. In the past, China's generic manufacturers exhibited numerous shortcomings, such as small-scale production, uneven quality, and outmoded production technology. For a long time, physicians and patients were skeptical of the quality of generic drugs, which hindered the industry's sustainable development and further increased the burden on the Chinese health system. Another is the lack of government regulation. In the last few decades, many drugs were approved without proper testing due to various reasons. The GCE functions not only as a quality test but also as a means of simplifying NVBP procedures (usually finished within 40 days) and improving the transparency of tendering since all drugs that pass the GCE are considered for pricing based on almost only the criterion of price.

Another significant process difference between TPF and NVBP is that the latter directly links pricing and procurement. Under TPF, the price of a generic drug is determined by the price of the branded product and the number of existing manufacturers when there is no specific or guaranteed procurement. However, for NVBP, the JPO releases detailed information about procurement (order quantity, period, location, etc.) before the bidding process. Thus, Canadian generic drug manufacturers need to make risk decisions with unclear order quantities and clear prices, whereas Chinese manufacturers enter into market competition with unclear prices but clear order quantities. Nonetheless, TPF is also associated with procurement from public programmes: generic drugs failing to match the pCPA’s Calculated Unit Price will be considered non-compliant and delisted from public drug plan formularies consequently.

We also believe that the NVBP represents a transfer of purchasing power from decentralised medical institutions, which are difficult to supervise, to a centralised organisation. As the main purchasing entities, the public medical institutions in the NVBP jurisdictions are explicitly required by the governments to commit to the entire volume they submitted after completing the price negotiation. Also, to ensure that the selected drugs receive priority use in hospitals, the governments have implemented several supporting policies. The NHSA incorporates the selected drugs into the national formulary and clarifies the new payment arrangements. In addition, the Nation Health Commission modifies the performance evaluation approaches of public hospitals with new incentive systems. For example, hospitals with priority use and guaranteed dosage of the selected drugs are preferred in the receipt of awards and funding under the public hospital reform background, whereas hospitals that failed to complete the tasks receive some administrative penalties, like reduced government financial subsidies or lower annual performance score [21]. Thus, due to the mission of hospitals, administrators of hospitals might pressure physicians to prioritise the use of the negotiated products. However, some deficiencies of NVBP have also gradually been revealed, such as the indirect prohibition of the use of other products in public hospitals, which might excessively interfere with the prescribing choice of physicians and affect the normal clinical needs of patients to a large degree. In Canada's decentralised health care system, such a transfer of purchasing power is unlikely to occur. Furthermore, the regulation on procurement is well-established. The balance between buyers and manufacturers, and federal and province/territory governments could be broken, if pCPA tried to centralise the purchasing power. TPF and NVBP processes summary is presented in **Table 1**.

**Table 1:** Overview of Tiered Pricing Framework and National Volume-Based Procurement

	Items	Tiered Pricing Framework	National Volume-Based Procurement
Structure	Macro context	Federal state	Unitary country
	Promoter	Province/territory governments (upstream)	Central government (downstream)
	Main parties involved	Intergovernmental institutions, alliances, provincial/territory government, pharmaceutical industry	National departments, alliances, public medical institutions, pharmaceutical industry
	Type of priced drugs	Generic drug	Mainly for generic drug
	Healthcare system reform objectives	Pan-Canadian system of drug coverage	Value-based strategic purchasing
		Structural reforms to improve efficiency	Increase health service prices and improve medical worker’s compensation plan
	Programme objectives	Lower the prices of generic drugs	Significant reduction in drug price and patients’ burden
		Increase coverage and price consistency across public drug plans	Reduction in transaction cost
			Improve the pharmaceutical industry ecology
			Improve the drug using
			Support the reform of public hospitals
			Explore the bulk purchasing mechanism
Process	Pricing mechanism	Tiered price scheme	Tendering
	Beginning	Manufacturer submits application	Government releases procurement information
	Entry criteria	No	Generics consistency evaluation
	Price cut limit	15 ~ 75%	0 ~ 100%
	Procurement	Pricing and procurement are not directly linked.	Pricing and procurement are directly linked.
		Overpriced generic drugs will be delisted from public drug plan formularies.	Transferring the purchasing power from the separative medical institute to a centralised organisation.
Outcome	Efficiency	748 kinds of generic drugs. About 98 generic drugs per year	345 drugs in total. About 86 drugs per year.
	Effective	53% on average (15 ~ 75%)	53% on average (0 ~ 98%)
	Savings	740 million CAD in savings annually	17 billion CAD savings annually
	Other outcomes	Generic drug price consistency	Release patients’ financial burden
		Accelerates generic entry in small markets	Motivated doctors to prescribe more affordable bidding winner drugs
		Protect interests of the opening-up-market manufacturer	

CAD – Canadian dollar

#### Outcome

##### Tiered Pricing Framework Outcomes

From its inception to July 2022, TPF assessed and priced 748 types of generic drugs, resulting in an average price reduction of 53%. According to the pCPA, as of April 2022, TPF has saved an annual total of 740 million Canadian dollars (CAD) [36]. In addition to cost savings, TPF has helped maintain consistency in generic drug pricing across the provinces/territories, contributing to improved health equity. However, it is not clear from the publicly available evidence whether TPF has led to an expansion of public drug coverage in Canada.

By comparing drug prices and market entry before and after the establishment of pCPA, TPF has been shown to accelerate the entry of generic drugs into small markets, creating the benefits of generic competition while avoiding the drawbacks of previously used price-cap regulations [27].

##### National Volume-Based Procurement Outcomes

As of the end of 2022, seven rounds of NVBP had been conducted, resulting in the selection of 294 drugs and an average price cut of 53% (ranging from 0 to 98%). According to the leader of NHSA, as of February 2022, the savings from NVBP had reached 260 billion RMB (approximately 51 billion CAD).

NVBP has successfully reduced drug prices, alleviated patients’ financial burdens, and improved accessibility [37,38]. NVBP has motivated doctors to prescribe more affordable bidding-winner drugs, instead expensive originator drugs [39]. However, total drug expenditures were not effectively controlled due to the increasing use of the drugs that have won the bidding process [40].

##### Overlapped drugs comparison

We compared the drug lists of NVBP and TPF and found 60 overlapping drugs as shown in **Table 2**. All price information was adjusted to the baseline year of 2021 using purchase power parity (PPP) and exchange rate data provided by the World Bank. The prices under NVBP were 37.03% (exchange rate) and 57.00% (PPP) of their TPF counterparts. However, given that China’s adjusted net national income per capita is only about 20% of Canada's in the most recent year, drug prices remain relatively expensive for the Chinese population, resulting in limited accessibility. Tablets had the lowest relative price, while tablets in extended-release form had the highest.

**Table 2:** Overview of overlapped drugs (n = 60)

Ingredient name	Dosage form	Strength	Standardised price of NVBP (CAD)*	Standardised price of TPF (CAD)*	Comparison by exchange rate†	Comparison by purchase power parity‡
Entecavir	Tablet	0.5 mg	0.04	5.5000	0.72%	1.11%
Adefovir dipivoxil	Tablet	10 mg	0.18	20.7067	0.88%	1.35%
Nifedipine	Tablet	20 mg	0.01	1.0702	1.07%	1.64%
Glimepiride	Tablet	1 mg	0.01	0.8186	1.29%	1.99%
Glimepiride	Tablet	2 mg	0.01	0.8908	1.43%	2.19%
Captopril	Tablet	25 mg	0.00	0.3052	1.44%	2.22%
Tenofovir disoproxil fumarate	Tablet	300 mg	0.08	4.5775	1.66%	2.56%
Leflunomide	Tablet	10 mg	0.10	2.9252	3.34%	5.14%
Ceftriaxone sodium	Powder for solution	1000 mg	0.49	14.0106	3.49%	5.37%
Ambrisentan	Tablet	5 mg	4.21	115.0584	3.66%	5.63%
Fluconazole	Tablet	50 mg	0.05	1.2083	3.88%	5.97%
Indapamide hemihydrate	Tablet	2.5 mg	0.01	0.2499	5.18%	7.98%
Levofloxacin	Tablet	250 mg	0.09	1.5892	5.66%	8.71%
Ceftriaxone sodium	Powder for solution	250 mg	0.26	4.4292	5.94%	9.14%
Cefprozil	Tablet	250 mg	0.15	1.7374	8.35%	12.85%
Cefuroxime	Tablet	250 mg	0.09	0.8868	10.00%	15.39%
Clindamycin hydrochloride	Capsule	150 mg	0.03	0.2281	11.26%	17.33%
Pioglitazone hydrochloride	Tablet	30 mg	0.10	0.8721	11.37%	17.50%
Pioglitazone hydrochloride	Tablet	15 mg	0.08	0.6225	12.99%	19.99%
Cetirizine hydrochloride	Tablet	10 mg	0.03	0.2082	13.44%	20.69%
Ceftazidime	Powder for solution	1000 mg	1.82	13.0052	14.02%	21.58%
Aripiprazole	Tablet	5 mg	0.13	0.9046	14.14%	21.76%
Sildenafil citrate	Tablet	25 mg	0.40	2.8062	14.42%	22.20%
linezolid	Tablet	600 mg	7.59	43.8011	17.33%	26.67%
Ticagrelor	Tablet	60 mg	0.19	1.0612	18.16%	27.96%
Lisinopril	Tablet	10 mg	0.05	0.2221	20.86%	32.10%
Levofloxacin	Tablet	500 mg	0.38	1.8110	21.26%	32.72%
Metoprolol tartrate	Tablet	50 mg	0.02	0.0831	23.27%	35.82%
Meropenem trihydrate	Powder for solution	500 mg	2.03	8.6359	23.49%	36.15%
Zoledronic acid	Solution	4 mg/5 ml	8.83	37.4082	23.60%	36.33%
Ticagrelor	Tablet	90 mg	0.28	1.1124	24.79%	38.15%
Candesartan cilexetil	Tablet	4 mg	0.05	0.2114	25.20%	38.79%
Montelukast sodium	Granules	4 mg	0.38	1.4906	25.34%	39.00%
Duloxetine hydrochloride	Capsule	60 mg	0.23	0.9148	25.51%	39.26%
Acarbose	Tablet	50 mg	0.04	0.1348	26.32%	40.51%
Oseltamivir phosphate	Capsule	75 mg	0.38	1.0393	36.52%	56.21%
Enalapril maleate	Tablet	10 mg	0.11	0.3045	36.85%	56.72%
Capecitabine	Tablet	500 mg	0.55	1.4829	37.23%	57.30%
Temozolomide	Capsule	100 mg	34.80	87.4648	39.79%	61.24%
Moxifloxacin hydrochloride	Tablet	400 mg	0.59	1.4261	41.45%	63.80%
Gefitinib	Tablet	250 mg	8.41	19.3732	43.39%	66.78%
Letrozole	Tablet	2.5 mg	0.70	1.5380	45.41%	69.88%
Apixaban	Tablet	2.5 mg	0.53	1.1473	46.43%	71.46%
Esomeprazole magnesium	Tablet	20 mg	0.25	0.5150	47.87%	73.67%
Temozolomide	Capsule	20 mg	9.46	17.4923	54.09%	83.26%
Voriconazole	Tablet	50 mg	4.02	7.1697	56.09%	86.33%
Escitalopram oxalate	Tablet	10 mg	0.82	1.4495	56.64%	87.18%
Losartan potassium	Tablet	50 mg	0.21	0.3609	58.50%	90.04%
Lacosamide	Tablet	50 mg	0.35	0.5911	58.73%	90.39%
Repaglinide	Tablet	1 mg	0.06	0.0942	61.14%	94.11%
Lurasidone hydrochloride	Tablet	40 mg	0.72	1.1471	63.15%	97.19%
Lacosamide	Tablet	100 mg	0.58	0.8194	71.11%	109.45%
Olmesartan medoxomil	Tablet	20 mg	0.21	0.2827	74.93%	115.33%
Abiraterone acetate	Tablet	250 mg	5.96	7.6563	77.81%	119.77%
Bicalutamide	Tablet	50 mg	1.62	1.9548	82.89%	127.57%
Quetiapine fumarate	Tablet er	50 mg	0.23	0.2342	100.33%	154.42%
Quetiapine fumarate	Tablet er	200 mg	0.66	0.6237	105.67%	162.64%
Gliclazide	Tablet er	30 mg	0.13	0.0872	151.21%	232.73%
Cefazolin sodium	Powder for solution	500 mg	1.30	0.8438	154.15%	237.26%
Cefazolin sodium	Powder for solution	1000 mg	2.39	1.2215	195.95%	301.59%
In total (tablet)	Tablet	N/A	N/A	N/A	28.45%	43.76%
In total (capsule)	Capsule	N/A	N/A	N/A	33.43%	51.46%
In total (powder for solution)	Powder for solution	N/A	N/A	N/A	66.17%	101.85%
In total (tablet er)	Tablet er	N/A	N/A	N/A	119.07%	183.26%
In total	N/A	N/A	N/A	N/A	37.03%	57.00%

TPF – Tiered Pricing Framework, CAD – Canadian dollar, NVBP – National Volume-Based Procurement, mg – milligramme, ml – millilitre, N/A – not applicable

*Discounted into 2021.

†Standardised price of NVBP drug/standardised price of TPF drug by exchange rate.

‡Standardised price of NVBP drug/standardised price of TPF drug by purchase power parity.

**Figure 3:**
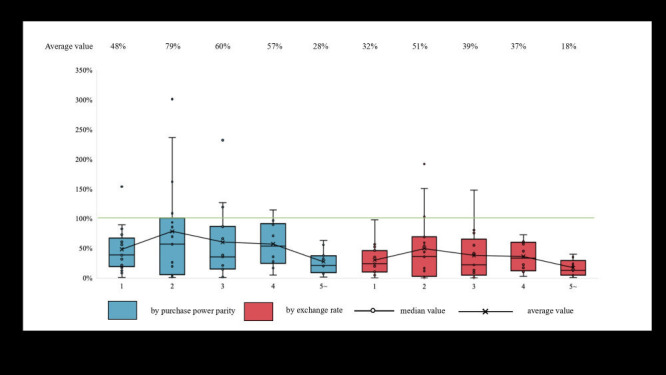
Overview of National Volume-Based Procurement (NVBP) and Tiered Pricing Framework (TPF) outcomes.

We also found that the number of winning manufacturers under NVBP affected relative prices, with better results achieved when there was a single or more than four winning manufacturers (**Figure 3**).

#### Outcome comparison

During the last seven and a half years, TPF assessed and priced 748 kinds of generic drugs, which amounts to an average of approximately 98 drugs per year. If we consider data since the implementation of NVBP in 2019, the number is 141 drugs per year. Since the implementation of NVBP in 2019, 345 drugs have been included in the programme, which equates to approximately 86 drugs per year. The efficiency of TPF is significantly higher than that of NVBP in recent years, possibly because the drug list and order size for tendering are determined by various government sectors, which slows down the pricing process. It's important to emphasise that the results of this comparison do not necessarily represent the ideal or maximum efficiency attainable by the two systems. Instead, they reflect the observed outcomes in practical implementation. The number of drugs assessed by TPF may primarily reflect pharmaceutical companies' willingness to participate. Their motivation is often driven by commercial benefits and market demand rather than inherent limitations of the TPF system itself. In contrast, the Chinese government plays a more proactive role in determining the number and types of drugs included in NVBP's tendering process. These decisions are typically based on factors such as the health care needs of the Chinese population, the availability of existing suppliers, and the prevalence of specific drugs within the market. These figures provide valuable insights into how TPF and NVBP have performed in real-world scenarios, but they should not be interpreted as definitive measures of the inherent capabilities of the systems. Both systems have their unique strengths and limitations, and their effectiveness can vary depending on specific contexts and evolving circumstances within the pharmaceutical market.

**Figure 4:**
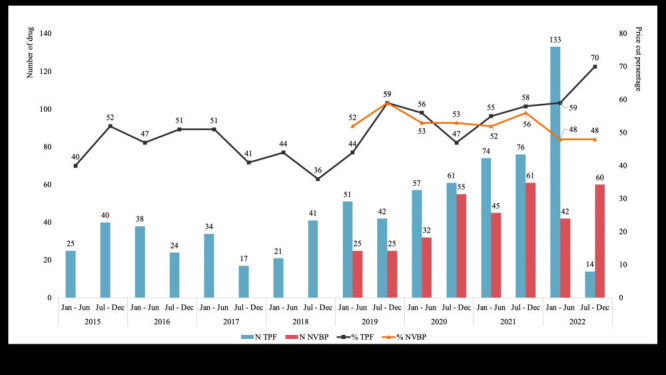
Price comparison of overlapped drugs by winner manufacturer numbers of National Volume-Based Procurement (NVBP).

TPF regulations stipulate that the price reduction should be between 15 and 75%, depending on the number of manufacturers. However, NVBP does not have any specific regulations on the percentage of price reduction, and the actual range in practice is between 0 and 98%, which is less restrictive than TPF. Since the beginning of pCPA, some experts question the rationality of TPF’s three-tiered pricing mechanism and believe that a 75% price cut is still too low for some generic drugs because production costs may be closer to 2 or 3% of the price of the brand-name drug [10,41]. In fact, in recent years, there are some arguments that Canada could consider tendering [42,43]. However, our results show that the effectiveness of NVBP and TPF is rather similar, with both programmes achieving an average price reduction of approximately 53%. Therefore, we cautiously assume adding more tiers or increasing the price cut percentage of TPF may not lead to a further reduction in drug prices. An overview of the information on NVBP and TPF is presented in **Figure 4**.

Both TPF and NVBP have achieved cost savings in drug spending through pricing mechanisms that leverage resources from various participants. Yet, we refrain from directly comparing these savings due to significant differences in population size, morbidity and mortality profiles, and other contextual factors. However, governments need to proceed with caution and consider the risks of emphasising the reduction of drug prices without a long-term plan for balancing the interests of multiple stakeholders. If the government is too focused on achieving a reduction in drug prices and fails to create an acceptable framework for guaranteeing sales volume to compensate for the revenue losses of manufacturers, generic manufacturers may have less incentive to introduce certain products into the market or postpone their introduction. The case of Apotex can explain why the price cut is as low as 15 or 25% when there is a single supplier in Canada [44]. Apotex developed a new formulation for Lipitor that it believed did not infringe Pfizer’s remaining patents. Before Pfizer sued Apotex for patent infringement, Apotex had spent many millions of dollars and managed to get approval from Health Canada. Once Apotex had cleared all the relevant hurdles, Pfizer did not object to other generic manufacturers entering the market [44]. In this case, Apotex, as the first generic manufacturer, bore almost all the risk of patent infringement, and the following generic manufacturers could share the benefits of opening up the market. As for NVBP, the majority of the listed suppliers are large domestic companies. Some multinational companies, such as Pfizer and Sanofi, also provided discounts of 10 to 30% at the beginning of the tendering activity, but some of them withdrew when faced with the incredibly low prices offered by Chinese drug industries. Those multinationals usually are less willing to compromise on a low price; thus, making it difficult for them to survive in such an environment. In reality, the prices of non-winning products also decreased after NVBP bidding to save the market [45].

Under the NVBP, a key characteristic is that once a company wins the bid with the lowest price, it can quickly capture a large portion of the market share. However, there are several risks associated with this phenomenon. In some cases, winning bidders are possibly not manufacturers with large-scale production capacity, which raises concerns about their ability to guarantee a sufficient drug supply and avoid shortages. Additionally, non-winning bidders may experience significant market share losses, which could potentially lead to bankruptcy. The high purchase volume associated with the NVBP once led to economic chaos in the Chinese pharmaceutical market. Companies participating in the NVBP bidding have gone to great lengths to win, causing drug prices to fall well beyond market expectations. The stock price of bidding loser companies, even some winning companies, tumbled about 15% on average [46,47]. This has created a paradox: the NVBP, which aimed to improve the pharmaceutical industry's ecology, has instead disrupted the market [48]. It is uncertain whether this kind of stock fluctuation will continue in the future, but the leading reason for it is likely the transfer of purchasing power, as previously mentioned. It is also concerning whether the huge purchase volume associated with the NVBP will lead to the monopolisation of some pharmaceutical enterprises with strong production capacity, as this would violate the programme’s original intention of achieving price reduction through competition. The era of high gross profits for generic drugs is over, and small enterprises without high-quality products, production capacity, and core competitiveness will be eliminated one after another [47]. Furthermore, the order size from well-developed provinces is typically larger than that from less-developed provinces, intensifying market competition. As a result, drug prices in more developed provinces are lower than those in less developed provinces, exacerbating health inequities and reducing drug accessibility.

Another significant difference between TPF and NVBP is that the latter requires many supporting policies to ensure that the contracts can be fulfilled. As mentioned earlier, TPF does not directly impact procurement, whereas specific contracts are signed between suppliers and public buyers after NVBP. The central and local governments implement many supporting policies to ensure that all these contracts can be fulfilled and to incentivise the use of drugs from winning bidders. These policies, on the one hand, interfere with the prescribing choices of physicians and, on the other hand, discourage over-prescription. **Table 1** provides a summary of the outcomes of TPF and NVBP.

The durability of NVBP and TPF may differ significantly. In terms of historical precedent, previous attempts like price caps have lasted for decades in Canada. Since TPF has been working well since its implementation, there is no reason to question its continuation. However, views about NVBP are conflicting. Some argue that NVBP should become a regular and normalised programme to achieve sustainable price reductions for generic drugs. On the other hand, some suggest that NVBP has fulfilled its historical mission and that the central government should delegate its centralised purchasing power to local governments [49]. It is difficult to determine which view is correct at this moment, but a pricing mechanism that provides long-term stability is needed in China. This mechanism could be NVBP or some other policy design. The primary characteristic of NVBP is a price reduction, and many people now take a 50% reduction in price for granted. The price of generic drugs cannot continuously decrease. The question is whether NVBP is still necessary if it achieves zero price reductions in the future.

It is also interesting to note that some people have discussed and studied the impact of TPF or NVBP on pharmaceutical innovation [48]. However, innovation encouragement is not one of the objectives of either programme, and we will not be discussing it in this study.

### The pan-Canadian Select Molecules

The pan-Canadian Select Molecules (pCSM) is a separate policy arrangement from the Tiered Pricing Framework, but it is still important for Canada’s access to affordable generic drugs. A comparison was made between pCSM and the NVBP, with 33 drugs overlapping under both programmes. Drug prices under NVBP were found to be higher than under pCSM: 67.38% (ranging from 9.31 to 217.44%) and 103.70% (ranging from 14.33 to 334.67%) of their pCSM counterparts, according to exchange rate and PPP, respectively. More details can be found in Appendix S4 in the **Online Supplementary Document**.

## DISCUSSION

This paper uses Donabedian’s SPO framework to conduct a comparative analysis of two pricing cases in different political settings. The study identifies major health care system reform objectives, pharmaceutical market maturity and history as key factors that shape policy choices in China and Canada. While both programmes aim to lower the prices of generic drugs, the macro health care system endows each programme with distinguished objectives. The study concludes that a tiered pricing scheme is feasible in regions with a stable and mature pharmaceutical market, typically seen in high-income countries, while tendering is more workable in low- and middle-income countries where the pharmaceutical market is weak and unstable with an obscure pricing mechanism. The primary difference between the tiered pricing scheme (TPF) and tendering (NVBP) is how purchasing power is transferred. TPF only sets prices and leaves the final procurement decision to each jurisdiction, whereas tendering combines orders from multiple jurisdictions to gain bargaining power. Although both TPF and NVBP achieve similar effectiveness in price reduction, NVBP requires massive supporting policies and has a deep involvement of the government and downstream policy design, which may impede its efficiency. The analysis of 60 overlapped drugs suggests that Chinese drugs are still relatively expensive, leading to more health inequity and worse accessibility. Single or more than four suppliers can achieve better results in NVBP tendering.

In this specific case, the structure and process do not operate in parallel but rather interact to influence the outcome. The structure defines the framework within which the process operates, providing the foundational settings and limiting the available choices. When considering the major objectives of health care system reform in Canada and China, there are various potential pathways beyond NVBP or TPF, such as price negotiation, reference pricing, price caps, and others [12]. However, the historical context and existing systems in each country constrain their choices to the current approaches. The Generics Consistency Evaluation is another example where the structure influences the process. The GCE plays a role in ensuring basic quality assurance for domestic drugs due to the poor quality of such drugs. In many other low- and middle-income countries, the subpar quality of domestic drugs similarly restricts their policy options for implementing rational pricing [50]. Similarly, the protection of the interests of generic drug manufacturers in open markets is a common process arrangement in high-income countries, driven by their mature and robust pharmaceutical markets and intellectual property systems [51]. All these unique process designs, influenced by the underlying structure, ultimately affect the outcome.

Indeed, the structure can directly impact the outcome as well. The Chinese market size is significantly larger than that of Canada, resulting in larger savings. Moreover, due to the differences in the drug access system, TPF primarily focuses on retail pharmacy prices, while NVBP emphasises public hospitals. Additionally, the majority of generic drugs sold in China are manufactured domestically, whereas, in Canada, most companies do not have domestic manufacturing capabilities for generic drugs. Consequently, the costs of generic drugs are likely to differ. However, with all these variations, it remains challenging to precisely measure the influence of the structural attributes on the outcome.

It's important to note that various strategies are employed for drug pricing, such as value-based pricing, price negotiation, reference pricing, and price caps [52-54]. In essence, drug pricing is a commercial activity carried out within the legitimate rights of its suppliers. Throughout this process, government entities, societal groups, media and various stakeholders exert pressure to achieve more affordable prices. Extensive global research indicates that the purchasing power held by public payers and market competition are two fundamental mechanisms for driving price reductions.

Regarding the former, suppliers with prices exceeding specific thresholds (determined through methods like reference pricing, price negotiation, cost-effectiveness analysis, or other strategies) may be excluded from public drug plans, effectively curbing overpricing. For example, the National Institute for Health and Care Excellence in the United Kingdom operates with an approximate 30 thousands Great Britain pounds (£) threshold and often receives submissions with costs hovering around £29 thousands per incremental cost-effectiveness ratio [55]. Similar scenarios have also occurred within tiered pricing frameworks.

In terms of market competition, the presence of multiple suppliers incentivises them to offer more affordable prices and capture a larger market share. For instance, mandatory substitution reforms in Sweden substantially reduced prices of locally sourced drugs [56]. In the United States, a study demonstrated that drug shortages had a more pronounced impact on the prices of drugs supplied by only three or fewer manufacturers, highlighting the detrimental effects of insufficient market competition [57].

In addition to the intricacies of pricing mechanisms, it is important to acknowledge the role of trust and ecosystem development within pharmaceutical pricing systems. While our primary focus remains the comparative analysis of China's NVBP and Canada's TPF, these concepts offer valuable insights into the dynamics of these systems. Trust, as a foundational element, fosters collaborative planning and negotiation between pharmaceutical companies and government bodies. In the context of competitive pricing mechanisms, this trust allows companies to engage in forward-looking strategies that extend beyond immediate cost considerations. Manufacturers can anticipate potential financial losses on certain medicines and compensate through other means, thereby contributing to the stability and sustainability of the pharmaceutical ecosystem.

Furthermore, we recognise the significance of understanding the landscape of pharmaceutical providers within each system. Unfortunately, obtaining precise and up-to-date information on the number of companies participating in these pricing systems proved challenging due to confidentiality and proprietary data concerns. Nevertheless, this contextual information could offer valuable insights into the market dynamics influencing pricing strategies.

As we delve into the comparative analysis of NVBP and TPF, we encourage readers to consider the overarching principles of trust, collaborative planning, and ecosystem development that underpin these systems. While these concepts may not be the primary focus of our study, they play a crucial role in shaping the pharmaceutical pricing landscape.

### Limitations

This comparative study examined the pharmaceutical pricing mechanisms in Canada and China, focusing on the Tendered Pricing Framework and the National Volume-Based Procurement system. It is essential to acknowledge that these systems are fundamentally different, making direct comparisons challenging. Our analysis provides a construct to assess how well they perform within their respective contexts, but these are essentially less-comparable schemes due to variations in health care systems, regulations, and contextual factors. Methodological difficulties arise when comparing pricing mechanisms across different countries, as diverse factors influence outcomes. Limited data availability, temporal dynamics, and contextual nuances further complicate such analyses. While our study offers valuable insights, it is crucial to interpret the findings within these limitations and avoid oversimplifying the complexities of these distinct pharmaceutical pricing systems. Researchers and policymakers should exercise caution when drawing policy implications and consider the unique circumstances of each health care system.

## CONCLUSIONS

Based on the analysis, we have formulated a series of recommendations for future actions that should be considered by policy makers in both China and Canada, as well as in other countries, where applicable. First, develop a clear and consistent working framework to guide negotiations in the aspects of both ensuring negotiation incentives and mandates for parties to meet their commitments and meanwhile protecting the autonomy and benefit of each jurisdiction. Access to pharmaceuticals is a multidimensional challenge that requires integrated policies and strategies with the engagement of all related parties. Experience in the two countries has shown that pricing mechanisms can involve lots of piecemeal interactive problems, a sophisticated system with a robust long-range plan may address these better. Second, strengthen rational drug selection and pricing mechanisms through the pursuit of patient-centred, value-informed and evidence-based health care. Lowering the price of generic drugs is not the whole story, particularly in the context of tendering. If pharmaceutical companies engage in collusion during bidding, participate in bid-rigging, or resort to cutthroat competition with excessively low prices to secure contracts, it may result in a situation where inferior products prevail, potentially leading to supply failures. A rational drug pricing mechanism can only be achieved by massive collaboration among the government, pharmaceutical manufacturers and the public. Third, involve multiple payers who want to be at the negotiation table, which not only could strengthen purchasing power, but also benefit more patients. Another important consideration is to avoid excluding potential drug suppliers from participating in the bidding process. China’s experience has shown that including as many manufacturers as possible can strengthen competition and lead to lower drug prices. A diversified supplier system can also help ensure a stable supply of drugs.

In conclusion, the study underscores the intricate nature of pricing mechanisms in the pharmaceutical market and emphasises the requirement for an integrated approach involving multiple stakeholders, rational drug selection, and pricing mechanisms that prioritise the cost of supply.

## Additional material


Online Supplementary Document
