# Supplementary material for: The pan-Canadian Tiered Pricing Framework and Chinese National Volume-Based Procurement: A comparative study using Donabedian’s structure-process-outcome framework
Source: J Glob Health. 2023 Nov 10;13:04137. doi: 10.7189/jogh.13.04137 (PMC10636597; doi:10.7189/jogh.13.04137)
Supplement: Online Supplementary Document [file jogh-13-04137-s001.pdf]

ONLINE SUPPLEMENTARY DOCUMENT

Title: The pan-Canadian Tiered Pricing Framework and Chinese National Volume-Based Procurement: A Comparative Study Using Donabedian's Structure-Process-Outcome Framework

Authors: Quan Wang, Siqi Liu, Zhijie Nie, Zheng Zhu, Yaqun Fu, Jiawei Zhang, Xia Wei, Li Yang, Xiaolin Wei

Address and posit for each author:

Quan Wang, MM, MSc[a, b]

Ph.D. student

Siqi Liu, MM, MSc[c, d]

Assistant Researcher

Zhijie Nie, MBBS[a]

Master student

Zheng Zhu, MBBS[a]

Master student

Yaqun Fu, MM, MSc[a]

Ph.D. student

Jiawei Zhang, MM[a]

Ph.D. student

Xia Wei, MM[e]

Ph.D. student

Li Yang, Ph.D.[a]

Professor

Xiaolin Wei, MBBS, PhD, FFPH(UK) [f]

Professor and Fellow of Canadian Academy of Health Sciences (FCAHS)

[a] *School of Public Health, Peking University, Beijing, China*

[b] *Brown School, Washington University in St. Louis, St. Louis, MO, U.S.*

[c] *Center of Health System and Policy, Institute of Medical Information & Library, Chinese Academy of Medical Sciences & Peking Union Medical College, Beijing, China*

[d] *Institute of Health Policy, Management, and Evaluation, Dalla Lana School of Public Health, University of Toronto, Toronto, Ontario, Canada,*

[e] *Department of Health Services Research and Policy, London School of Hygiene & Tropical Medicine, London, UK*

[f] *Dalla Lana School of Public Health, University of Toronto, Toronto, Ontario, Canada*

Appendix S1 The Information Collection Process

Table S1 Search strategies to identify studies

Database searched: MEDLINE

Date: March 8, 2023

Limits: till March 8, 2023

#	Searches	Results
1	(Tiered Pricing Framework[Title/Abstract]) OR (pan-Canadian Pharmaceutical Alliance[Title/Abstract])	17
2	((Volume-based Procurement[Title/Abstract]) OR ("4+7"[Title])) AND (China[MeSH Terms])	18
3	#1 OR #2	35

Database searched: Web of Science

Date: March 8, 2023

Limits: till March 8, 2023

#	Searches	Results
1	(TS=(Tiered Pricing Framework)) OR TS=(pan-Canadian Pharmaceutical Alliance)	258
2	((TS=(Volume-based Procurement)) OR TI=(4+7)) AND TS=(China)	64
3	#1 OR #2	322

Database searched: Embase

Date: March 8, 2023

Limits: till March 8, 2023

#	Searches	Results
1	(tiered AND ('pricing'/exp OR pricing) AND framework:ab,ti OR 'pan canadian') AND pharmaceutical AND alliance:ab,ti	38
2	('volume based' AND procurement:ab,ti OR '4+7':ti) AND ('china'/exp OR 'china')	215
3	#1 OR #2	253

Database searched: Grey Matters Checklist

Date: March 8, 2023

Limits: till March 8, 2023

#	Databases	Searches	Results

1	Bandolier Knowledge	Tiered Pricing Framework (Match All Words)	0
2	Bandolier Knowledge	pan-Canadian Pharmaceutical Alliance (Match All Words)	0
3	Bandolier Knowledge	Volume-based Procurement China (Match All Words)	0
4	McMaster University, McMaster Health Forum	pan-Canadian Pharmaceutical Alliance OR Tiered Pricing Framework	0
5	Canadian Agency for Drugs and Technologies in Health (CADTH)	Tiered Pricing Framework OR pan-Canadian Pharmaceutical Alliance	201
6	Latin-American and Caribbean Center on Health Sciences Information (LILACS)	Tiered Pricing Framework [Words] or pan-Canadian Pharmaceutical Alliance [Words] Tittle, abstract, subject	0

Table S2 PICO of data extraction

	TPF	NVBP
Population	Patients in Canada who use prescription drugs	Patients in China who use NVBP bidding-winner drugs
Intervention	Tiered Pricing Framework of the pan-Canadian Pharmaceutical Alliance	National Volume-Based Procurement
Comparison	N/A	
Outcome	Drug pricing and affordability, drug access and availability, drug utilization and adherence, health outcomes, healthcare costs, patient and healthcare provider satisfaction, equity	
Study Design	Any type of quantitative or qualitative studies evaluating the implementation or impact	

Appendix S2 Tiered Pricing Framework Process

The TPF process begins with application from a generic manufacturer, and there are two kinds of manufacturer activities that may trigger pricing or re-pricing: market entry and market exit.

Market entry:

1) For a generic drug to be listed on any public drug plan formulary, manufacturers are required to submit an application to pan-Canadian Pharmaceutical Alliance Office (pCPAO).
2) The pCPAO will verify whether the Tiered Pricing Framework applies to the candidate drug and determine if the product is a single, dual, or multi-source. For single-source products (Tier 1), if there is a product listing agreement (PLA) or pricing agreement for the brand in any jurisdiction, the product will be priced at 75% of the brand reference price; otherwise, it will be priced at 85% (products of single source will be reassessed after two years). Dual sources (Tier 2) and multi-sources (Tier 3) products will be priced at 50% and 25% of the brand reference price, respectively.
3) The pCPAO will determine the appropriate price tier and advise the manufacturer and all participating jurisdictions of the assessment results.
4) The manufacturer submits its application to individual jurisdictions for listing on a provincial formulary. Jurisdictions retain sole discretion over the final coverage decision of products listed on public drug plan formularies. Once a TPF Pricing Confirmation Form has been received and assessed by the pCPAO, all competitors are expected to adjust their price to match the pCPA's Calculated Unit Price in all jurisdictions, including where the product was listed prior to pCPA assessment.

Market exit:

1) The generic manufacturer or pCPA jurisdiction(s) submit a "Market Exit Notification" to the pCPAO.
2) The pCPAO confirms with the generic manufacturer that it has no intention to re-enter the Canadian market and outlines additional relevant details.
3) The pCPA conducts evaluation in 6 months to make sure the generic manufacturer meets standards to exit the market: no wholesaler supply and sales less than Generic Category Threshold (0.5% to 2% of current category volume, based on market share history).
4) The pCPAO processes Market Exit Application.
5) The pCPAO will notify jurisdictions, the Submitting Manufacturer (and competitors if there is a change in tier) of the assessed tier and price. All competitors currently listed will be given the opportunity to adjust their prices to match the assessed price point

established through the TPF during jurisdictions' next regular/scheduled formulary updates.

As showed above, no activities from the province/territory governments, generic manufacturers, or the pCPA itself will directly affect the price of generic drugs under TPF, and the only mediator is TPF tier. In other words, only those activities lead to TPF tier change will trigger the price change. A central process for price increase applications is being contemplated and currently the details is still unclear. It's worth noting that a brand reference price increase will not result in an increase in the price of generic drugs. This is because the brand reference price is established when the first generic drug is assessed through the TPF, and this price will be used for all future assessments.

Appendix S3 National Volume-Based Procurement Process

Currently, the NVBP drug list is determined by Chinese governments but there are no clear and publicly available rules on what triggers NVBP covering a specific kind of drug. As declared by the leader of NHIB, following conditions are requirements: having no less than 2 manufacturers in the market, being covered by the National Health Insurance List (NHIL), being clinically necessary, having huge market size, and high spending. In nature, NVBP is a type of tendering conducted by Chinese central government twice a year, covering different drugs each time.

Unlike TPF, the NVBP is not open for all generic drugs: only those that have passed Generics Consistency Evaluation (GCE) can participate in the tendering process. The GCE tests the quality and efficacy of China's domestically produced generic drugs in comparison to the corresponding brand-name version. If a drug has passed the GCE, it means it is identical in terms of quality and efficacy with the brand reference drug. Therefore, during the bidding process of NVBP, the JPO need only consider the prices of drug bidders, without their quality or efficacy.

The NVBP links pricing (by tendering), purchasing and using:

1) Drugs are selected after the expert consultation and JPO approval;
2) Public medical institutions estimate the total purchase volume of the selected drugs based on 60% to 70% of the total annual drug consumption in the past;
3) The JPO is in charge of the bidding process and determines the winning drugs. Price is the most important criterion, so the manufacturer(s) with the lowest price(s) are usually selected as the winner(s). The JPO may also consider the credit records of bidders, but there is no public information indicating that any manufacturer is excluded because of a bad record.
4) After the final price established, public medical institutions or their local public procurement agency are expected to sign contracts and purchase drugs with the final supplier independently.

The entire procurement process will be regulated by both central and local governments to ensure that suppliers and buyers comply with the contract terms. Typically, 30% of the total costs will be directly transferred from local health insurance bureaus to medical facilities to alleviate their financial burden. The medical facilities are obligated to pay the full amount within 30 days after receiving the entire drug supply.

Appendix S4 Comparison Result of NVBP and pCSM Drugs

Table S2 Overview of overlapped drugs between NVBP and pCSM (N=33)

Ingredient name	Dosage form	Price of NVBP (RMB)	Price of TPF (CAD)	Comparison by exchange rate*	Comparison by purchase power parity†
CANDESARTAN CILEXETIL	tablet	0.2281	0.2636	22.69%	34.92%
DONEPEZIL HCL	tablet	0.4586	0.8936	38.26%	58.88%
SIMVASTATIN	tablet	0.2501	0.1369	10.75%	16.54%
SOLIFENACIN SUCCINATE	tablet	0.3041	3.3679	217.44%	334.67%
MYCOPHENOLATE MOFETIL	capsule	0.3712	1.2874	66.14%	101.80%
MYCOPHENOLATE MOFETIL	tablet	0.7423	1.6000	41.11%	63.27%
ANASTROZOLE1MG	tablet	0.9522	2.1927	44.77%	68.91%
CELECOXIB	capsule	0.2558	0.4879	37.08%	57.08%
FINASTERIDE	tablet	0.3506	0.3796	21.05%	32.40%
MEMANTINE HCL	tablet	0.4420	0.7881	34.67%	53.36%
METFORMIN HCL	tablet	0.0247	0.0610	48.02%	73.90%
OLANZAPINE ODT	tablet	0.3574	0.8614	46.86%	72.13%
OMEPRAZOLE	capsule	0.2287	0.1536	13.06%	20.10%
SERTRALINE HCL	tablet	0.3032	1.4429	92.53%	142.41%
VALSARTAN	tablet	0.2211	0.1360	11.96%	18.41%
GABAPENTIN	capsule	0.0416	0.1252	58.52%	90.06%
PRAMIPEXOLE DIHYDROCHLORIDE	tablet	0.1950	0.6567	65.48%	100.78%
PREGABALIN	capsule	0.3007	1.8191	117.62%	181.03%
TELMISARTAN	tablet	0.2161	0.4990	44.90%	69.10%

TERBINAFINE HCL	tablet	0.7714	0.8607	21.69%	33.39%
VALSARTAN/HCTZ	tablet	0.2213	1.8650	163.86%	252.19%
AMLODIPINE BESYLATE	tablet	0.1343	0.0622	9.31%	14.33%
ATORVASTATIN CALCIUM	tablet	0.1743	0.1879	21.68%	33.36%
ATORVASTATIN CALCIUM	tablet	0.2179	0.5486	50.63%	77.92%
CLOPIDOGREL BISULFATE	tablet	0.2631	2.6533	202.78%	312.11%
IRBESARTAN	tablet	0.2281	0.1933	17.04%	26.23%
IRBESARTAN	tablet	0.2281	0.3401	29.98%	46.14%
IRBESARTAN/HCTZ	tablet	0.2281	1.0512	92.67%	142.63%
LEVETIRACETAM	tablet	0.3210	2.3948	150.01%	230.89%
MONTELUKAST SODIUM	tablet	0.4231	3.8340	182.21%	280.45%
OLANZAPINE	tablet	0.7088	4.9662	140.89%	216.84%
PAROXETINE HCL	tablet	0.3250	1.5883	98.27%	151.25%
RISPERIDONE	tablet	0.2031	0.0963	9.53%	14.67%

[*]: Standardized price of NVBP drug / standardized price of TPF drug by exchange rate

[†]: Standardized price of NVBP drug / standardized price of TPF drug by purchase power parity
